# 5-Methyl-3,6,7,8a-tetra­hydro-2*H*-diimidazo[1,2-*c*:1′,2′-*e*]pyrido[1,2-*a*][1,3,5]triazin-5-ium iodide

**DOI:** 10.1107/S1600536810019495

**Published:** 2010-05-29

**Authors:** Aleksandra Wasilewska, Franciszek Sączewski, Maria Gdaniec

**Affiliations:** a Department of Chemical Technology of Drugs, Medical University of Gdańsk, 80-416 Gdańsk, Poland; b Faculty of Chemistry, Adam Mickiewicz University, 60-780 Poznań, Poland

## Abstract

The structure of the title compound, C_12_H_16_N_5_^+^·I^−^, shows that the methyl­ation reaction with CH_3_I occurred at the imine N atom at position 5 of the 3,6,7,8a-tetra­hydro-2*H*-diimidazo[1,2-*c*:1′,2′-*e*]pyrido[1,2-*a*][1,3,5]triazine system. In the cation,  the *sp* ^3^-hybridized C atom belonging to the fused dihydro­pyrine and dihydro-1,3,5-triazine rings deviates by 0.514 (3) Å from the best plane defined by the remaining cationic non-H atoms. The fused dihydro­pyridine and dihydro-1,3,5-triazine rings are each in a half-chair conformation with the *sp* ^3^-hybridized C atom as a flap. The iodide anion is 3.573 (2) Å from the methyl­ated N atom and exhibits five short C—H⋯I^−^ contacts with distances less than 3.16 Å. The structure has been determined from a non-merohedral twin with twin law [−1 0 0 0 − 1 0 0.115 0 1], minor domain = 0.1559 (12).

## Related literature

For the synthesis and data reported earlier for the title compound, see: Sączewski & Foks (1981 ▶). For the programs used to derive the twin law, see: Cooper *et al.*(2002 ▶); Farrugia (1999 ▶). 
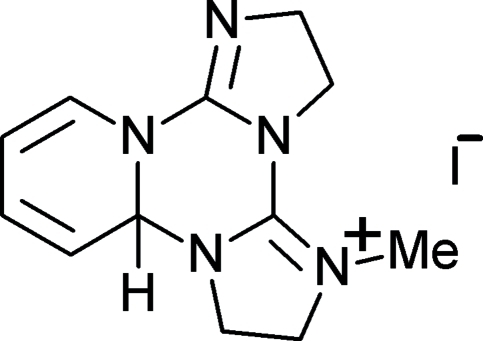


## Experimental

### 

#### Crystal data

C_12_H_16_N_5_^+^·I^−^
*M* *_r_* = 357.20
Monoclinic, 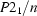

*a* = 7.6299 (2) Å
*b* = 15.3939 (4) Å
*c* = 11.4503 (3) Å
β = 92.204 (2)°
*V* = 1343.89 (6) Å^3^
*Z* = 4
Mo *K*α radiation
μ = 2.37 mm^−1^
*T* = 100 K
0.2 × 0.2 × 0.1 mm

#### Data collection

Oxford Diffraction Xcalibur-E CCD diffractometer
Absorption correction: multi-scan (*CrysAlis PRO*; Oxford Diffraction, 2009 ▶) *T*_min_ = 0.496, *T*_max_ = 0.789
25955 measured reflections
4406 independent reflections
4018 reflections with *I* > 2σ(*I*)
*R*_int_ = 0.026

#### Refinement

*R*[*F* ^2^ > 2σ(*F* ^2^)] = 0.029
*wR*(*F* ^2^) = 0.070
*S* = 1.20
4406 reflections
165 parameters
H-atom parameters constrained
Δρ_max_ = 1.74 e Å^−3^
Δρ_min_ = −1.24 e Å^−3^

### 

Data collection: *CrysAlis PRO* (Oxford Diffraction, 2009 ▶); cell refinement: *CrysAlis PRO*; data reduction: *CrysAlis PRO*; program(s) used to solve structure: *SHELXS97* (Sheldrick, 2008 ▶); program(s) used to refine structure: *SHELXL97* (Sheldrick, 2008 ▶) and *WinGX* (Farrugia, 1999 ▶); molecular graphics: *ORTEP-3 for Windows* (Farrugia, 1997 ▶); software used to prepare material for publication: *SHELXL97*.

## Supplementary Material

Crystal structure: contains datablocks global, I. DOI: 10.1107/S1600536810019495/tk2676sup1.cif

Structure factors: contains datablocks I. DOI: 10.1107/S1600536810019495/tk2676Isup2.hkl

Additional supplementary materials:  crystallographic information; 3D view; checkCIF report

## Figures and Tables

**Table 1:** Hydrogen-bond geometry (Å, °)

*D*—H⋯*A*	*D*—H	H⋯*A*	*D*⋯*A*	*D*—H⋯*A*
C3—H3*B*⋯I1^i^	0.99	3.15	4.007 (3)	145
C6—H6*A*⋯I1^ii^	0.99	3.05	4.010 (3)	163
C9—H9⋯I1^iii^	0.95	3.08	4.025 (3)	172
C12—H12⋯I1^iv^	0.95	3.14	3.887 (3)	137
C17—H17*C*⋯I1^i^	0.98	3.16	4.025 (3)	148

Symmetry codes: (i) 
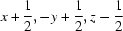
; (ii) 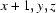; (iii) 
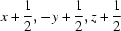
; (iv) 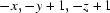.

**Fig. 1.**
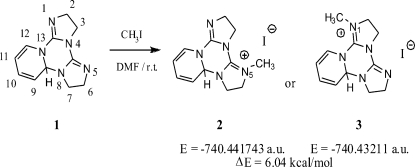
Reaction of 2,3,6,7,8a,13-hexahydropyrido[1,2-a]diimidazo[1',2',-c:1",2"-e]-1,3,5-triazine (1) with methyl iodide.

**Fig. 2.**
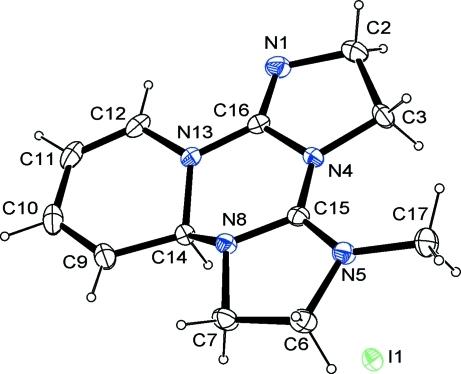
Asymmetric unit for the title salt with displacement ellipsoids shown at the 50% probability level.

**Fig. 3.**
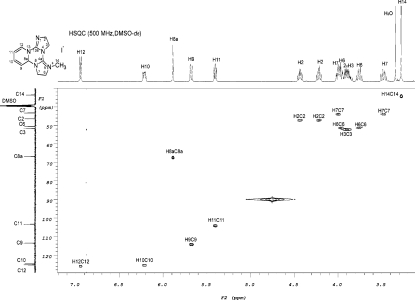
HSQC spectrum of the title compound 2

**Fig. 4.**
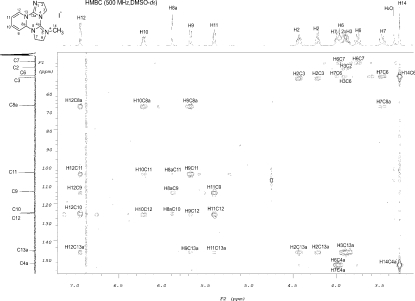
HMBC spectrum of the title compound 2

## supplementary crystallographic information

### Comment

Biguanide derivatives are known to possess diverse biological activities, including antidiabetic, antibacterial, germicidic, antiviral and antimalarial. On the other hand, quaternary ammonium salts constitute a well known class of bacteriostatic agents. Therefore, we have decided to synthesize some *N*-alkylated cyclic biguanide derivatives for biological testing, based on the previously described procedure (Sączewski & Foks, 1981) which consists in the reaction of 2,3,6,7,8a,13-hexahydropyrido[1,2-a]diimidazo[1',2'-c:1'',2''-e]-1,3,5-triazine (1) with an alkyl halide. As shown in Fig. 1, the course of the reaction of 1 with methyl iodide has not been established and two products, 2 or 3, arising from either N1 or N5 alkylation have been proposed. In this work, based on X-ray structure analysis (Fig. 2) and hetero-correlation NMR experiments (HSQC and HMBC), the structure of the title compound (2) is determined unambiguously. Regioselectivity of *N*-alkylation of the cyclic biguanide derivative 1 could not be predicted on the basis of calculated electrostatic potential and charge distribution. The structure of the N5 alkylated product 2 was also confirmed by 2D NMR spectroscopic data. Thus, assignment of signals observed in ^1^H and ^13^C-NMR spectra was possible using HSQC spectrum (see numbering scheme in Fig. 3). The crucial signals of quaternary carbon atoms C13a and C4a were found at 145.7 and 152.6 p.p.m., respectively. 3-Bond correlation from the latter carbon to a singlet of three protons at 3.26 p.p.m. observed in the HMBC spectrum (Fig. 4) indicated the placement of methyl group at the N5 nitrogen atom.

### Experimental

1D and 2D NMR spectra were recorded on a Varian Unity 500 spectrometer. The title compound was prepared according to the previously described procedure (Sączewski & Foks, 1981); m.p. 492–494 K (decomp.); ^1^H NMR (500 MHz, DMSO-*d_6_*, see Fig. 3 for numbering scheme) δ_H _6.94 (1*H*, d, *J *= 7.2 Hz, H12), 6.22–6.19 (1*H*, ddd, *J=*9.8, 6.8, ~1 Hz, H10), 5.86 (1*H*, t, *J *= 1 Hz, H8a), 5.67 (1*H*, dd, *J* = 9.8, ~1 Hz, H9), 5.41–5.38 (1*H*, dd, *J *= 7.2, 6.8 Hz, H11), 4.42 (1*H*, dt, *J =* 9.3, 6.8 Hz, H2), 4.20 (1*H*, dt, *J =* 9.3, 6.8 Hz, H2), 4.01–3.83 (4*H*, m, H7, H6, 2xH3),3.78–3.71 (1*H*, m, H6), 3.49–3.43 (1*H*, m, H7), 3.26 (3*H*, s, CH_3_); ^13^C NMR (125 MHz, DMSO-*d_6_*, see Fig. 3 for numbering scheme) δ_C_ 152.6 (C4a), 145.7 (C13a), 125.4 (C12), 124.8 (C10), 113.6 (C9), 103.4 (C11), 67.0 (C8a), 51.7 (C3), 50.7 (C6), 46.6 (C2), 43.3 (C7), 33.7 (C14); IR (KBr, cm^–1^): 3090, 3079, 3025, 2936, 2880, 1684, 1661, 1550, 1429, 1321, 1301, 1186, 677.

### Refinement

The twin matrix, -1 0 0/0 - 1 0/0.115 0 1, corresponding to 180° rotation about [0 0 1] direct lattice direction has been determined with the program ROTAX (Cooper *et al.*, 2002). For the refinement with the *SHELXL97* program (Sheldrick, 2008), the reflection data file was prepared in the HKLF 5 format using the 'Make HKLF5' function of the *WinGX* program (Farrugia, 1999). The overlapping reflections and those belonging to only one twin domain are used in the refinement (HKLF 5 format of *SHELXL97*). Those which were excluded, 132 reflections, are partial overlaps which could not be integrated properly at the data processing stage. The BASF parameter refined at 0.1559 (12). The H atoms bonded to C atoms were placed at calculated positions, with C—H = 0.95–1.00 Å, and refined as riding on their parent atoms, with *U*_iso_(H) = *x U*_eq_(C), where *x* = 1.5 for the H atoms from the methyl group and *x* = 1.2 for the remaining H atoms. The maximum and minimum residual electron-density peaks of 1.74 and -1.24 eÅ^-3^ were located 0.72 Å and 1.84 Å from H6B and I1 atoms, respectively.

### Crystal data

**Table:**

C_12_H_16_N_5_^+^·I^−^	*F*(000) = 704
*M**_r_* = 357.20	*D*_x_ = 1.765 Mg m^−^^3^
Monoclinic, *P*2_1_/*n*	Mo *K*α radiation, λ = 0.71073 Å
Hall symbol: -P 2yn	Cell parameters from 19213 reflections
*a* = 7.6299 (2) Å	θ = 2.7–32.3°
*b* = 15.3939 (4) Å	µ = 2.37 mm^−^^1^
*c* = 11.4503 (3) Å	*T* = 100 K
β = 92.204 (2)°	Block, colourless
*V* = 1343.89 (6) Å^3^	0.2 × 0.2 × 0.1 mm
*Z* = 4	

### Data collection

**Table:**

Oxford Diffraction Xcalibur-E CCD diffractometer	4406 independent reflections
Radiation source: fine-focus sealed tube	4018 reflections with *I* > 2σ(*I*)
graphite	*R*_int_ = 0.026
ω scan	θ_max_ = 32.4°, θ_min_ = 4.1°
Absorption correction: multi-scan (*CrysAlis PRO*; Oxford Diffraction, 2009)	*h* = −9→11
*T*_min_ = 0.496, *T*_max_ = 0.789	*k* = −23→23
25955 measured reflections	*l* = 0→16

### Refinement

**Table:**

Refinement on *F*^2^	Primary atom site location: structure-invariant direct methods
Least-squares matrix: full	Secondary atom site location: difference Fourier map
*R*[*F*^2^ > 2σ(*F*^2^)] = 0.029	Hydrogen site location: inferred from neighbouring sites
*wR*(*F*^2^) = 0.070	H-atom parameters constrained
*S* = 1.20	*w* = 1/[σ^2^(*F*_o_^2^) + (0.0196*P*)^2^ + 3.1405*P*] where *P* = (*F*_o_^2^ + 2*F*_c_^2^)/3
4406 reflections	(Δ/σ)_max_ = 0.001
165 parameters	Δρ_max_ = 1.74 e Å^−^^3^
0 restraints	Δρ_min_ = −1.24 e Å^−^^3^

### Special details

**Table:**

Geometry. All esds (except the esd in the dihedral angle between two l.s. planes) are estimated using the full covariance matrix. The cell esds are taken into account individually in the estimation of esds in distances, angles and torsion angles; correlations between esds in cell parameters are only used when they are defined by crystal symmetry. An approximate (isotropic) treatment of cell esds is used for estimating esds involving l.s. planes.
Refinement. Refinement of *F*^2^ against ALL reflections. The weighted *R*-factor wR and goodness of fit *S* are based on *F*^2^, conventional *R*-factors *R* are based on *F*, with *F* set to zero for negative *F*^2^. The threshold expression of *F*^2^ > σ(*F*^2^) is used only for calculating *R*-factors(gt) etc. and is not relevant to the choice of reflections for refinement. *R*-factors based on *F*^2^ are statistically about twice as large as those based on *F*, and *R*- factors based on ALL data will be even larger.

### Fractional atomic coordinates and isotropic or equivalent isotropic displacement parameters (Å^2^)

**Table:**

	*x*	*y*	*z*	*U*_iso_*/*U*_eq_	
I1	0.04305 (2)	0.194494 (11)	0.473423 (14)	0.01657 (5)	
N1	0.1806 (3)	0.53774 (16)	0.3251 (2)	0.0209 (5)	
C2	0.2057 (4)	0.4944 (2)	0.2120 (2)	0.0222 (5)	
H2A	0.2910	0.5272	0.1660	0.027*	
H2B	0.0931	0.4914	0.1663	0.027*	
C3	0.2755 (4)	0.40179 (19)	0.2385 (2)	0.0206 (5)	
H3A	0.1869	0.3569	0.2173	0.025*	
H3B	0.3846	0.3898	0.1973	0.025*	
N4	0.3083 (3)	0.40681 (15)	0.36610 (19)	0.0155 (4)	
N5	0.4690 (3)	0.27675 (15)	0.4159 (2)	0.0162 (4)	
C6	0.5326 (4)	0.23510 (18)	0.5250 (2)	0.0189 (5)	
H6A	0.6623	0.2316	0.5289	0.023*	
H6B	0.4837	0.1759	0.5322	0.023*	
C7	0.4659 (4)	0.29506 (18)	0.6205 (2)	0.0201 (5)	
H7A	0.3677	0.2679	0.6613	0.024*	
H7B	0.5609	0.3097	0.6785	0.024*	
N8	0.4068 (3)	0.37245 (14)	0.55467 (19)	0.0147 (4)	
C9	0.3144 (4)	0.4560 (2)	0.7238 (2)	0.0202 (5)	
H9	0.3752	0.4175	0.7759	0.024*	
C10	0.2718 (4)	0.5354 (2)	0.7605 (3)	0.0237 (6)	
H10	0.2938	0.5510	0.8400	0.028*	
C11	0.1922 (4)	0.59810 (19)	0.6798 (3)	0.0237 (6)	
H11	0.1462	0.6510	0.7082	0.028*	
C12	0.1842 (4)	0.58081 (18)	0.5651 (3)	0.0197 (5)	
H12	0.1363	0.6226	0.5118	0.024*	
N13	0.2459 (3)	0.50171 (14)	0.5233 (2)	0.0162 (4)	
C14	0.2675 (3)	0.42666 (17)	0.6013 (2)	0.0151 (4)	
H14	0.1556	0.3928	0.6010	0.018*	
C15	0.3916 (3)	0.35088 (16)	0.4392 (2)	0.0136 (4)	
C16	0.2403 (3)	0.48697 (17)	0.4045 (2)	0.0156 (5)	
C17	0.4863 (4)	0.2302 (2)	0.3064 (3)	0.0242 (6)	
H17A	0.3854	0.1916	0.2934	0.036*	
H17B	0.5944	0.1957	0.3102	0.036*	
H17C	0.4909	0.2719	0.2420	0.036*	

### Atomic displacement parameters (Å^2^)

**Table:**

	*U*^11^	*U*^22^	*U*^33^	*U*^12^	*U*^13^	*U*^23^
I1	0.01712 (8)	0.01865 (8)	0.01406 (7)	0.00055 (6)	0.00212 (5)	−0.00050 (6)
N1	0.0239 (12)	0.0181 (11)	0.0207 (11)	0.0010 (9)	−0.0007 (9)	0.0040 (9)
C2	0.0251 (14)	0.0244 (14)	0.0170 (12)	0.0007 (11)	−0.0009 (10)	0.0062 (11)
C3	0.0271 (15)	0.0233 (14)	0.0114 (11)	0.0015 (11)	−0.0001 (9)	0.0007 (10)
N4	0.0198 (11)	0.0150 (10)	0.0117 (9)	0.0004 (8)	0.0010 (7)	0.0016 (8)
N5	0.0174 (11)	0.0158 (9)	0.0155 (9)	0.0029 (8)	0.0033 (8)	0.0009 (8)
C6	0.0189 (13)	0.0187 (12)	0.0191 (12)	0.0056 (10)	0.0012 (9)	0.0035 (9)
C7	0.0245 (13)	0.0194 (12)	0.0161 (11)	0.0044 (10)	−0.0008 (9)	0.0045 (9)
N8	0.0157 (10)	0.0150 (9)	0.0134 (9)	0.0007 (8)	−0.0001 (7)	0.0017 (7)
C9	0.0213 (13)	0.0242 (13)	0.0152 (11)	−0.0016 (10)	0.0003 (9)	−0.0024 (10)
C10	0.0223 (14)	0.0297 (15)	0.0191 (13)	−0.0052 (11)	0.0023 (10)	−0.0092 (11)
C11	0.0241 (15)	0.0162 (12)	0.0312 (15)	−0.0029 (10)	0.0048 (11)	−0.0088 (11)
C12	0.0178 (13)	0.0126 (11)	0.0289 (14)	−0.0021 (9)	0.0045 (10)	−0.0026 (10)
N13	0.0212 (11)	0.0111 (9)	0.0164 (10)	0.0001 (8)	0.0001 (8)	−0.0002 (8)
C14	0.0166 (12)	0.0152 (11)	0.0135 (11)	−0.0006 (9)	0.0001 (8)	−0.0001 (9)
C15	0.0113 (11)	0.0147 (11)	0.0148 (10)	−0.0024 (8)	0.0022 (8)	0.0012 (8)
C16	0.0157 (12)	0.0130 (11)	0.0181 (11)	−0.0021 (9)	0.0012 (9)	0.0011 (9)
C17	0.0316 (16)	0.0213 (13)	0.0201 (12)	0.0047 (11)	0.0075 (11)	−0.0047 (10)

### Geometric parameters (Å, °)

**Table:**

N1—C16	1.270 (3)	C7—H7B	0.9900
N1—C2	1.476 (4)	N8—C15	1.364 (3)
C2—C3	1.547 (4)	N8—C14	1.468 (3)
C2—H2A	0.9900	C9—C10	1.336 (4)
C2—H2B	0.9900	C9—C14	1.504 (4)
C3—N4	1.475 (3)	C9—H9	0.9500
C3—H3A	0.9900	C10—C11	1.453 (5)
C3—H3B	0.9900	C10—H10	0.9500
N4—C15	1.344 (3)	C11—C12	1.340 (4)
N4—C16	1.415 (3)	C11—H11	0.9500
N5—C15	1.317 (3)	C12—N13	1.397 (3)
N5—C17	1.455 (4)	C12—H12	0.9500
N5—C6	1.470 (3)	N13—C16	1.378 (3)
C6—C7	1.533 (4)	N13—C14	1.466 (3)
C6—H6A	0.9900	C14—H14	1.0000
C6—H6B	0.9900	C17—H17A	0.9800
C7—N8	1.471 (3)	C17—H17B	0.9800
C7—H7A	0.9900	C17—H17C	0.9800
			
C16—N1—C2	107.1 (2)	C10—C9—C14	121.1 (3)
N1—C2—C3	107.4 (2)	C10—C9—H9	119.4
N1—C2—H2A	110.2	C14—C9—H9	119.4
C3—C2—H2A	110.2	C9—C10—C11	120.5 (3)
N1—C2—H2B	110.2	C9—C10—H10	119.7
C3—C2—H2B	110.2	C11—C10—H10	119.7
H2A—C2—H2B	108.5	C12—C11—C10	119.6 (3)
N4—C3—C2	101.0 (2)	C12—C11—H11	120.2
N4—C3—H3A	111.6	C10—C11—H11	120.2
C2—C3—H3A	111.6	C11—C12—N13	120.4 (3)
N4—C3—H3B	111.6	C11—C12—H12	119.8
C2—C3—H3B	111.6	N13—C12—H12	119.8
H3A—C3—H3B	109.4	C16—N13—C12	118.9 (2)
C15—N4—C16	122.4 (2)	C16—N13—C14	118.1 (2)
C15—N4—C3	129.9 (2)	C12—N13—C14	120.6 (2)
C16—N4—C3	107.6 (2)	N13—C14—N8	107.0 (2)
C15—N5—C17	131.1 (2)	N13—C14—C9	110.4 (2)
C15—N5—C6	110.0 (2)	N8—C14—C9	111.1 (2)
C17—N5—C6	118.7 (2)	N13—C14—H14	109.4
N5—C6—C7	103.6 (2)	N8—C14—H14	109.4
N5—C6—H6A	111.0	C9—C14—H14	109.4
C7—C6—H6A	111.0	N5—C15—N4	129.4 (2)
N5—C6—H6B	111.0	N5—C15—N8	112.7 (2)
C7—C6—H6B	111.0	N4—C15—N8	117.9 (2)
H6A—C6—H6B	109.0	N1—C16—N13	127.0 (3)
N8—C7—C6	103.0 (2)	N1—C16—N4	116.1 (2)
N8—C7—H7A	111.2	N13—C16—N4	116.9 (2)
C6—C7—H7A	111.2	N5—C17—H17A	109.5
N8—C7—H7B	111.2	N5—C17—H17B	109.5
C6—C7—H7B	111.2	H17A—C17—H17B	109.5
H7A—C7—H7B	109.1	N5—C17—H17C	109.5
C15—N8—C14	117.1 (2)	H17A—C17—H17C	109.5
C15—N8—C7	108.2 (2)	H17B—C17—H17C	109.5
C14—N8—C7	119.1 (2)		

### Hydrogen-bond geometry (Å, °)

**Table:**

*D*—H···*A*	*D*—H	H···*A*	*D*···*A*	*D*—H···*A*
C3—H3B···I1^i^	0.99	3.15	4.007 (3)	145
C6—H6A···I1^ii^	0.99	3.05	4.010 (3)	163
C9—H9···I1^iii^	0.95	3.08	4.025 (3)	172
C12—H12···I1^iv^	0.95	3.14	3.887 (3)	137
C17—H17C···I1^i^	0.98	3.16	4.025 (3)	148

Symmetry codes: (i) *x*+1/2, −*y*+1/2, *z*−1/2; (ii) *x*+1, *y*, *z*; (iii) *x*+1/2, −*y*+1/2, *z*+1/2; (iv) −*x*, −*y*+1, −*z*+1.
